# Effects of Cotton Stalk and Sugar Beet Pulp Silage on Growth Performance, Serum Biochemistry, Rumen Fermentation, and Microbiota in Suffolk Ram Lambs

**DOI:** 10.3390/biology15161399

**Published:** 2026-08-15

**Authors:** Nuerminamu Aihemaiti, Zhanpeng Wang, Yongkuo Li, Linhai Song, Yili Liu, Tong Li, Buweiaizhaer Maimaitim, Wanping Ren, Wei Shao, Liang Yang

**Affiliations:** 1 College of Animal Science, Xinjiang Agricultural University, Urumqi 830052, China; 17590816304@163.com (N.A.);; 2 Manasi County Animal Disease Prevention and Control Center, Changji 832200, China; 3 Xinjiang Laboratory of Nutrition for Dairy and Meat Herbivores, Urumqi 830052, China

**Keywords:** silage, ruminant nutrition, cotton stalk, sugar beet pulp, Suffolk sheep, rumen fermentation, rumen microbiota

## Abstract

This study evaluated the effects of replacing whole-plant corn silage with cotton stalk and sugar beet pulp mixed silage at 0, 30, 60, or 90% on growth performance, serum parameters, and rumen characteristics in Suffolk ram lambs, addressing the lack of systematic research on moderate-to-high replacement ratios in sheep diets. Eighty-four 4-month-old ram lambs (32.36 ± 2.92 kg) were assigned to four treatments (3 pens/treatment, 7 lambs/pen) for a 120-day trial after a 15-day adaptation. Based on the growth performance and serum parameter results, the control and 60% replacement groups were selected for further analysis of rumen fermentation and bacterial microbiota via 16S rRNA gene sequencing. Compared with the control, the 60% replacement group showed increased final body weight (+15.7%, *p* < 0.01), daily weight gain (+38.4%, *p* < 0.01), and dry matter intake (+8.6%, *p* < 0.05), and improved feed-to-gain ratio (F:G) (*p* < 0.01). Serum immunoglobulin A and G increased (*p* < 0.05), whereas tumor necrosis factor-α and interleukin-1β decreased (*p* < 0.05) in the 60% and 90% replacement groups. Serum MDA was significantly lower in the 90% CSBPS group than in the control at d 90 (*p* < 0.05), with no significant differences among groups at d 120. Rumen isobutyric and valeric acid concentrations were greater in the 60% replacement group than in the control (*p* < 0.05), accompanied by enrichment of fiber-degrading bacteria including the Lachnospiraceae NK3A20 group. Replacing 60% of whole-plant corn silage with cotton stalk and sugar beet pulp silage was associated with improved growth performance, enhanced immune function and antioxidant status, and optimized rumen fermentation in Suffolk ram lambs under the conditions of this study.

## Simple Summary

High-cost and limited supply of corn silage hinder the sustainable development of sheep breeding. Cotton stalks and sugar beet pulp are abundant, underutilized agricultural by-products in Xinjiang with great feeding potential. This 120-day trial was performed on 84 Suffolk ram lambs to assess the effects of gradient substitution (0%, 30%, 60%, 90%) of whole-plant corn silage with cotton stalk–sugar beet pulp mixed silage (CSBPS). The results showed that 60% CSBPS substitution demonstrated favorable effects on growth performance and feed efficiency under the present experimental conditions, enhancing serum immunity and antioxidant capacity and optimizing rumen fermentation via elevated beneficial branched-chain volatile fatty acids. Moreover, this treatment enriched core fiber-degrading rumen microbes (e.g., Lachnospiraceae NK3A20 group and F082 family), potentially improving fiber degradation and host physiological homeostasis. Collectively, CSBPS can serve as a sustainable corn silage alternative for lamb fattening. This work provides a practical approach for agricultural by-product recycling and green, efficient mutton sheep production.

## 1. Introduction

China is a major producer and consumer of meat sheep, and a stable supply of roughage resources is fundamental to sustainable industry development [1]. Xinjiang, as a primary cotton and sugar beet producing region, generates substantial quantities of agricultural by-products, including cotton stalks and sugar beet pulp. However, cotton stalks are highly lignified and pose palatability and safety challenges when fed directly, whereas sugar beet pulp has high moisture content and is prone to spoilage, limiting its value as fresh feed [2]. These constraints contribute to regional shortages of high-quality roughage and elevated feed costs, which have hindered efforts to reduce production expenses in local meat sheep farming [3]. Fermenting a mixture of cotton stalks and sugar beet pulp to produce micro-silage can effectively improve palatability, enhance fermentation quality, increase nutritional value, and enable long-term storage [4], offering a practical strategy for utilizing these abundant by-products. Preliminary studies have investigated the effects of mixing ratios on the fermentation quality and nutritional value of cotton stalk and sugar beet pulp silage. Lu et al. [4] evaluated the in vitro gas production characteristics and fermentation parameters of mixed fermentation products at different proportions, providing a basis for optimizing the combination ratio. Yang et al. [5] further reported that different proportions of beet pulp and cotton stalk fermentation significantly influenced nutrient composition and fermentation parameters. Based on these findings, a 50:50 dry matter ratio was selected for the present study as a balanced approach to improve the fermentation quality of cotton stalks while utilizing the moisture and fermentable carbohydrates provided by sugar beet pulp. However, systematic in vivo evaluations of the resulting mixed silage as a replacement for conventional corn silage in lamb diets remain lacking. Based on these findings, the present study adopted a 5:5 (50:50) dry matter ratio for CSBPS preparation.

Suffolk ram lambs are a key sire breed for crossbreeding and genetic improvement of meat sheep in China, and their growth performance and health status directly influence economic returns [6]. Previous studies have demonstrated that low-proportion fermented cotton stalks can significantly reduce feed-to-gain ratio, improve growth performance, and increase the abundance of Prevotella in the rumen of sheep [7]. Prevotella is one of the most abundant genera in the rumen and plays a key role in starch and protein degradation, making it an important indicator of rumen fermentation efficiency and dietary adaptation in sheep. Similarly, Dadashi et al. [8] reported that the nutritional value of silage made from a mixture of sugar beet pulp and wheat straw is comparable to that of corn silage, with no significant difference in dry matter digestibility. However, these investigations have largely focused on single-ingredient applications or low substitution ratios (≤30%), and the systematic effects of moderate-to-high replacement ratios (60% or more) on rumen fermentation patterns, microbial community structure, and host immune and antioxidant status remain poorly understood. This knowledge gap restricts the efficient and safe utilization of these locally abundant by-products in intensive sheep production. Previous studies have investigated various inclusion levels of fermented agricultural by-products and their effects on blood metabolites, rumen fermentation, and microbial communities [7,8]. However, these studies have largely focused on low substitution levels (≤30%), and comprehensive evaluations of moderate-to-high replacement ratios (≥60%) on host immune function, antioxidant status, and rumen microbiota remain limited.

Therefore, the objective of this study was to evaluate the effects of replacing whole-plant corn silage with cotton stalk and sugar beet pulp mixed silage at 0, 30, 60, or 90% on growth performance, serum biochemical parameters, immune and antioxidant status, rumen fermentation characteristics, and bacterial microbiota in Suffolk ram lambs. These findings are expected to provide a scientific basis for optimizing the utilization of locally abundant agricultural by-products in sheep feeding systems and contribute to sustainable feed resource development in cotton and sugar beet producing regions.

## 2. Materials and Methods

### 2.1. Ethics Statement

All animal procedures were approved by the Experimental Animal Welfare Ethics Committee of Xinjiang Agricultural University (Approval No.: xjau-aw-2025-0217, date of approval: 5 February 2025) and conducted in accordance with the National Regulations on the Administration of Laboratory Animals and the Guiding Opinions on the Humane Treatment of Laboratory Animals issued by the Ministry of Science and Technology of China. The experiment followed the ARRIVE guidelines 2.0 (https://arriveguidelines.org accessed on 1 January 2025). The experiment was carried out from May to December 2025 at Xin’ao Animal Husbandry Co., Ltd. in Manas County, Xinjiang, China.

### 2.2. Preparation of Cotton Stalk and Sugar Beet Pulp Mixed Silage

The CSBPS was prepared by shredding cotton stalks to approximately 1 cm in length and mixing them with fresh sugar beet pulp (moisture content: 75 ± 3%) at a dry matter ratio of 5:5. A specialized fermentation compound inoculant for cotton stalks (total viable count ≥ 1 × 10^8^ CFU/g) was uniformly sprayed at 0.1% of the fresh weight, followed by the addition of urea (0.2%) and salt (0.05%). The moisture content was adjusted to 60–65%, and the mixture was compacted to a density of approximately 900 kg/m^3^, sealed with polyethylene film, and subjected to anaerobic fermentation at 25–30 °C for 120 days. The use of agricultural by-products such as white mulberry (Morus alba) pomace and leaves as alternative ruminant feed sources has recently been evaluated for fermentation kinetics and digestibility [9], supporting the broader applicability of micro-silage strategies for regional feed resource development.

### 2.3. Experimental Design and Animals

A total of 84 healthy Suffolk ram lambs, approximately 4 months old with an initial body weight of 32.36 ± 2.92 kg, were used in this study. The lambs were randomly allocated to four dietary treatment groups in a completely randomized design, with 21 lambs per treatment (3 replicate pens per treatment, 7 lambs per pen). The four treatment groups were as follows: CK (control group): fed a basal diet containing 21% whole-plant corn silage (DM basis); ML: fed a diet in which 30% of the whole-plant corn silage (DM basis) was replaced with CSBPS; MT: fed a diet in which 60% of the whole-plant corn silage (DM basis) was replaced with CSBPS; MC: fed a diet in which 90% of the whole-plant corn silage (DM basis) was replaced with CSBPS. The basal diet was formulated according to the Chinese Feeding Standards for Meat Sheep (NY/T 816-2021) [9]. Ten diets were formulated to have similar crude protein and metabolizable energy levels by adjusting corn and soybean meal proportions. The ingredient and chemical compositions of the experimental diets are presented in Table 1. The actual fresh-weight additions of CSBPS are shown in Table 1, with the basis for replacement ratio calculations detailed in the table footnote.

### 2.4. Feeding Management

All sheep were dewormed and adaptively fed the basal diet for 15 d before the trial. The animals were housed in group pens (7 lambs per pen, 3 pens per treatment) within a semi-open steel structure barn. Each pen was equipped with automatic water drinkers, feed troughs, and slatted floors. Indoor temperature was maintained at 14–22 °C and did not exceed 25 °C in summer; relative humidity was controlled at 55–60% and did not exceed 70%. During summer months, evaporative cooling pads and fans were operated when indoor temperature exceeded 25 °C to alleviate heat stress. A total mixed ration (TMR) was offered twice daily at 0900 and 1900, with ad libitum access to feed and water. Feed troughs and waterers were cleaned daily. Pens were thoroughly cleaned weekly; floors and troughs were rinsed with clean water and allowed to air-dry. Pens were disinfected every two weeks by spraying with a 10% povidone-iodine solution diluted 1:800, after sheep were temporarily moved to an adjacent pen. Health status was monitored daily throughout the trial.

### 2.5. Chemical Analysis of Feeds

Feed samples were dried at 65 °C for 48 h and ground to pass through a 1-mm screen. Dry matter (DM) was determined by oven drying at 105 °C for 24 h [10]. Crude protein (CP) was analyzed using the Kjeldahl method (N × 6.25; method 984.13) [11]. Ether extract (EE) was determined by Soxhlet extraction with petroleum ether [12]. Neutral detergent fiber (NDF) was assayed with heat-stable α-amylase and expressed exclusive of residual ash (aNDFom), and acid detergent fiber (ADF) was expressed exclusive of residual ash (ADFom), according to Van Soest et al. [13]. Crude ash was determined by combustion at 550 °C for 6 h [11]. Gross energy (GE) was determined by bomb calorimetry according to GB/T 45104-2024 [14], and metabolizable energy (ME) of the experimental diets was calculated using the NRC (2007) equations based on digestible nutrient concentrations [15]. The results are shown in Table 2.

### 2.6. Growth Performance Measurements

On d 0 and d 120 of the experiment, all experimental sheep were weighed on an empty stomach (fasted for 12 h) before morning feeding. Net weight gain and daily weight gain were calculated as follows:

Net weight gain (kg) = Final weight − Initial weight

Daily weight gain (g/d) = (Final weight − Initial weight)/Number of experimental days

Daily dry matter intake was accurately recorded during the trial, and leftover feed was collected and weighed the following morning. Daily dry matter intake was calculated per pen and divided by the number of animals in that pen to obtain the average daily dry matter intake per sheep:

Average daily dry matter intake (kg/d) = (Total feed provided − Total leftover feed)/(Number of trial days × Number of animals in the pen)

The feed-to-gain ratio (F:G) was calculated as follows:

F:G = Total dry matter intake/Total weight gain

### 2.7. Measurement of Serum Biochemical, Immunological, and Antioxidant Parameters

On d 0, 30, 60, 90, and 120, prior to morning feeding, blood samples (10 mL) were collected from the jugular vein of all lambs in each pen. Blood samples were allowed to clot at room temperature for 1 h and then centrifuged at 3000 rpm for 15 min to separate the serum. Serum samples were aliquoted into 2 mL centrifuge tubes and stored at −80 °C until analysis. All serum samples were sent to the Beijing Sino-UK Institute of Biological Technology (Beijing 100081, China) for centralized testing. Serum biochemical parameters, including total protein (TP), albumin (ALB), globulin (GLB), total cholesterol (TC), triglycerides (TG), high-density lipoprotein cholesterol (HDL-C), low-density lipoprotein cholesterol (LDL-C), creatinine (Cr), blood urea nitrogen (BUN), and glucose (GLU), were measured using colorimetric methods with commercially available kits (China National Biotec Group, Beijing, China) on a Mindray BS-420 fully automated biochemical analyzer (Shenzhen Mindray Medical Electronics Co., Ltd., Shenzhen, China). Immunological and cytokine parameters, including immunoglobulin A (IgA), immunoglobulin G (IgG), immunoglobulin M (IgM), tumor necrosis factor-α (TNF-α), interleukin-1β (IL-1β), and interleukin-4 (IL-4), were determined using enzyme-linked immunosorbent assay (ELISA) kits (Beijing Huaying Biotechnology Research Institute, Beijing, China) following the manufacturer‘s instructions. Optical density was measured using a Huawede Lang DR-200BS microplate reader (Wuxi Huawede Lang Instrument Co., Ltd., Wuxi, Jiangsu, China). Antioxidant parameters, including superoxide dismutase (SOD), malondialdehyde (MDA), glutathione peroxidase (GSH-Px), and total antioxidant capacity (T-AOC), were assayed using colorimetric kits (Beijing Huaying Biotechnology Research Institute) according to the manufacturer’s protocols. Absorbance was read using the same Huawede Lang DR-200BS microplate reader (Huawede Lang Instrument Co., Ltd., Wuxi, Jiangsu, China).

### 2.8. Rumen Fluid Sampling and Analysis

To explore the mechanisms underlying the observed differences in growth performance and host physiological responses, the control (CK) and 60% CSBPS replacement (MT) groups were selected for rumen fermentation and microbiota analysis.

#### 2.8.1. Rumen Fluid Collection

Six lambs per group (two randomly selected from each of the three pens) were fasted for 12 h before slaughter. The sheep were uniformly transported to the slaughterhouse (transport time approximately 25 min) for slaughter. Lambs were stunned with a non-penetrating captive bolt stunner and exsanguinated in compliance with the requirements of GB/T 42304-2023. All efforts were made to minimize suffering. The procedure followed the guidelines of the American Veterinary Medical Association [16] and the Chinese National Standard GB/T 42304-2023 [17]. Immediately after slaughter, rumen fluid was collected by aspirating approximately 50 mL of rumen contents using a sterile syringe. The rumen fluid samples were filtered through four layers of gauze, and the pH was immediately measured using a portable precision pH meter (HI981036, Hanna Instruments, Cluj-Napoca, Romania; accuracy 0.01). The filtrate was aliquoted into sterile centrifuge tubes, flash-frozen in liquid nitrogen, and transferred to −80 °C for subsequent analyses.

#### 2.8.2. Volatile Fatty Acid Analysis

Volatile fatty acids (VFAs) in rumen fluid were determined by gas chromatography. Briefly, 1 mL of filtered rumen fluid was mixed with 0.2 mL of 25% (*w*/*v*) metaphosphoric acid solution containing 2-ethylbutyric acid as an internal standard (final concentration 2 mmol/L), vortexed, and centrifuged at 12,000× *g* for 10 min. The supernatant was filtered through a 0.22 μm membrane and analyzed using a gas chromatograph (Agilent 7890B; Agilent Technologies, Santa Clara, CA, USA) equipped with a flame ionization detector (FID). A capillary column (30 m × 0.25 mm × 0.25 μm, DB-FFAP) was used, with high-purity nitrogen as the carrier gas at 1.0 mL/min. The injector and detector temperatures were 250 °C and 300 °C, respectively. The temperature program was: 100 °C for 1 min, ramped at 10 °C/min to 180 °C, then at 20 °C/min to 240 °C and held for 3 min. Injection volume was 1 μL with a split ratio of 10:1. Quantification was performed using the internal standard method with standard solutions of acetic, propionic, butyric, isobutyric, isovaleric, valeric, and caproic acids. The linear range was 0.1–20 mmol/L (R^2^ > 0.99). Intra- and inter-day precision relative standard deviations were <5%, and recovery rates ranged from 95% to 105%. Total VFA was calculated as the sum of individual VFA concentrations.

#### 2.8.3. 16S rRNA Gene Sequencing of Rumen Microorganisms

Total DNA was extracted from rumen fluid samples using the CTAB method. The V4 region of the bacterial 16S rRNA gene was amplified using primers 515F (5′-GTGCCAGCMGCCGCGGTAA-3′) and 806R (5′-GGACTACHVGGGTWTCTAAT-3′). After purification, PCR products were used for library construction with the NEB Next Ultra II DNA Library Prep Kit (New England Biolabs, Ipswich, MA, USA) and sequenced on the Illumina NovaSeq 6000 platform (Illumina, Inc., San Diego, CA, USA).

Sequencing data were demultiplexed, and paired-end reads were assembled using FLASH v1.2.11. Quality filtering was performed using fastp v0.23.1, and chimeric sequences were removed by alignment against the Silva v138.1 database using the UCHIME algorithm. Operational taxonomic units (OTUs) were clustered at 97% similarity using Uparse v7.0.1001, and taxonomic annotation was performed using the mothur algorithm against the Silva 138.1 database. Alpha diversity indices (Chao1, Shannon, Simpson) were calculated using QIIME v1.9.1, and beta diversity analysis (PCoA) was performed based on UniFrac distance. LEfSe (LDA > 4) was used to identify differentially abundant taxa between groups. PERMANOVA (permutational multivariate analysis of variance) with 999 permutations based on Bray–Curtis distance was used to test for significant differences in rumen bacterial community structure between CK and MT groups. Phylum names in figures and text are presented according to the GTDB taxonomy (e.g., *Bacillota* for Firmicutes, *Pseudomonadota* for Proteobacteria).

### 2.9. Statistical Analysis

All data were analyzed using SPSS 27.0. The experimental unit was the pen (n = 3 pens/treatment) for growth performance, dry matter intake, and serum parameters. Growth performance, dry matter intake, feed-to-gain ratio, and rumen fermentation were analyzed using one-way ANOVA followed by Tukey’s HSD test. For serum parameters measured repeatedly at five time points (d 0, 30, 60, 90, 120) on the same animals, data were analyzed using repeated-measures ANOVA with treatment as the between-subjects factor and time as the within-subjects factor; the treatment × time interaction was tested, and Bonferroni correction was applied for multiple comparisons. Rumen fermentation and microbial alpha diversity between CK and MT were compared by independent-samples *t*-test. For rumen microbiota, LEfSe with LDA > 4 was used to identify differentially abundant taxa. Spearman correlation analysis was performed as an exploratory screen to examine relationships between rumen microbial genera and host parameters; uncorrected *p*-values are shown. Normality was assessed using the Shapiro–Wilk test, and homogeneity of variance was assessed using Levene’s test. Data that met these assumptions were analyzed using parametric tests. For TNF-α, one-way ANCOVA with the d 0 value as a covariate was performed at each subsequent time point to account for significant baseline differences among groups. All data are presented as mean ± SEM. Significance was declared at *p* < 0.05, and highly significant differences at *p* < 0.01.

## 3. Results

### 3.1. Growth Performance

As shown in Table 3, final body weight and net weight gain in the MT and ML groups were significantly higher than those in the CK group (*p* < 0.01), and were significantly higher than those in the MC group (*p* < 0.01). Daily weight gain in the MT group (250.93 g/d) and the ML group (240.90 g/d) was significantly higher than that in the CK group (181.32 g/d) and the MC group (204.50 g/d) (*p* < 0.01). No significant difference was observed between the MT and ML groups (*p* > 0.01). The MT group had the highest average daily dry matter intake (1.65 kg/d), which was significantly higher than that of the CK group (*p* < 0.01) and the ML group (*p* < 0.01). The feed-to-gain ratio in the CK and MC groups was significantly higher than that in the ML and MT groups (*p* < 0.01). No significant difference was observed between the ML and MT groups (*p* > 0.05). Orthogonal polynomial contrast analysis revealed a significant linear trend for ADG across the increasing CSBPS substitution levels (*p* < 0.001), indicating a general dose-dependent improvement in growth performance. The quadratic trend approached but did not reach statistical significance (*p* = 0.051). A significant cubic trend was also detected (*p* = 0.037), suggesting a more complex response pattern rather than a simple unimodal curve with a single peak at 60%.

### 3.2. Serum Biochemical Parameters

As shown in Table 4, no significant differences were observed in TP, ALB, TC, TG, LDL-C, and BUN among the treatment groups at any time point (*p* > 0.05). On d 60, GLB content in the MC group was significantly higher than that in the CK group (*p* < 0.05). HDL-C content in the MT and MC groups was significantly higher than that in the ML group (*p* < 0.05). On d 120, creatinine levels in the CK group were significantly higher than those in the ML group (*p* < 0.05). The MT and MC groups showed a decreasing trend but the differences did not reach statistical significance. On d 60, glucose levels in the CK group were significantly higher than those in the ML group (*p* < 0.05).

### 3.3. Serum Immunological Parameters

As shown in Table 5, on d 30 and 90, IgA levels in the MT and MC groups were significantly higher than those in the CK group (*p* < 0.01) and the ML group (*p* < 0.05). No significant differences were observed between the MT and MC groups (*p* > 0.05). On d 30, IgG levels in the MC group were significantly higher than those in the CK group (*p* < 0.01). IgG levels in the ML and MT groups were significantly higher than those in the CK group (*p* < 0.05). On d 60, IgG levels in the MC group were significantly higher than those in the CK group (*p* < 0.05). The MT and ML groups showed an increasing trend but the differences did not reach statistical significance. On d 90, IgG levels in the MC group were significantly higher than those in the CK and ML groups (*p* < 0.05). No significant difference was observed between the MT group and the CK group (*p* > 0.05). On d 120, IgG levels in the MC and MT groups were significantly higher than those in the CK group (*p* < 0.01 and *p* < 0.05, respectively).

On d 30, IgM levels in the MC group were significantly higher than those in the CK group (*p* < 0.05). On d 90 and 120, IgM levels in the MC group were significantly higher than those in the CK group (*p* < 0.01).

On d 0, TNF-α levels in the CK group were significantly higher than those in the other groups (*p* < 0.01), indicating baseline imbalance. After adjusting for the d 0 baseline by ANCOVA, TNF-α levels showed a significant linear decrease with increasing CSBPS substitution at d 30, 60, 90, and 120 (*p* < 0.05). From d 30 to d 120, TNF-α levels in the treatment groups remained lower than those in the CK group, with the MC group showing the greatest reduction (18.4% lower than CK at d 120, *p* < 0.05). Orthogonal polynomial contrasts further confirmed a significant linear trend across substitution levels at d 30 (*p* < 0.001), d 60 (*p* = 0.021), d 90 (*p* = 0.012), and d 120 (*p* = 0.036), with no significant quadratic trends detected. On d 30, IL-1β levels in the CK and ML groups were significantly higher than those in the MC group (*p* < 0.05). No significant differences were observed among the CK, ML, and MT groups (*p* > 0.05). On d 120, IL-1β levels in the CK group were significantly higher than those in all experimental groups (*p* < 0.01). IL-1β levels in the ML group were significantly higher than those in the MT and MC groups (*p* < 0.05).

On d 60 and 90, IL-4 levels in the MT and MC groups were significantly higher than those in the CK and ML groups (*p* < 0.05 and *p* < 0.01, respectively). On d 120, IL-4 levels in the MC and MT groups were significantly higher than those in the CK and ML groups (*p* < 0.01 and *p* < 0.05, respectively). IL-4 levels in the MC group were higher than those in the MT group.

### 3.4. Serum Antioxidant Parameters

As shown in Table 6, SOD activity exhibited an increasing trend with increasing replacement ratio and experimental duration. From d 30 to d 120, SOD activity in all experimental groups was significantly higher than that in the CK group (*p* < 0.01 and *p* < 0.05). On d 90, MDA content in the CK group was significantly higher than that in the MC group (*p* < 0.05). On d 120, no significant differences were observed among groups (*p* > 0.05). GSH-Px activity increased significantly with increasing replacement ratio and duration (*p* < 0.05). The CK group was significantly lower than all experimental groups (*p* < 0.01). On d 60, GSH-Px activity in the ML group was significantly lower than that in the other groups (*p* < 0.01). On d 120, GSH-Px activity in the CK group was significantly lower than that in all experimental groups (*p* < 0.01).

### 3.5. Rumen Fermentation Parameters

Based on comprehensive growth performance and serum biochemical, immunological, and antioxidant indicators, the MT group exhibited superior overall performance. Therefore, the CK and MT groups were selected for slaughter and sampling to determine rumen fermentation characteristics. As shown in Table 7, isobutyric acid and valeric acid contents in the MT group were significantly higher than those in the CK group (*p* < 0.05). No significant differences were observed between the two groups for other indicators (*p* > 0.05). However, the MT group showed a trend toward higher levels of acetic acid, propionic acid, butyric acid, and total VFA compared to the CK group.

### 3.6. Rumen Microbiota

#### 3.6.1. Sequencing Depth and Alpha Diversity

**Figure 1:**
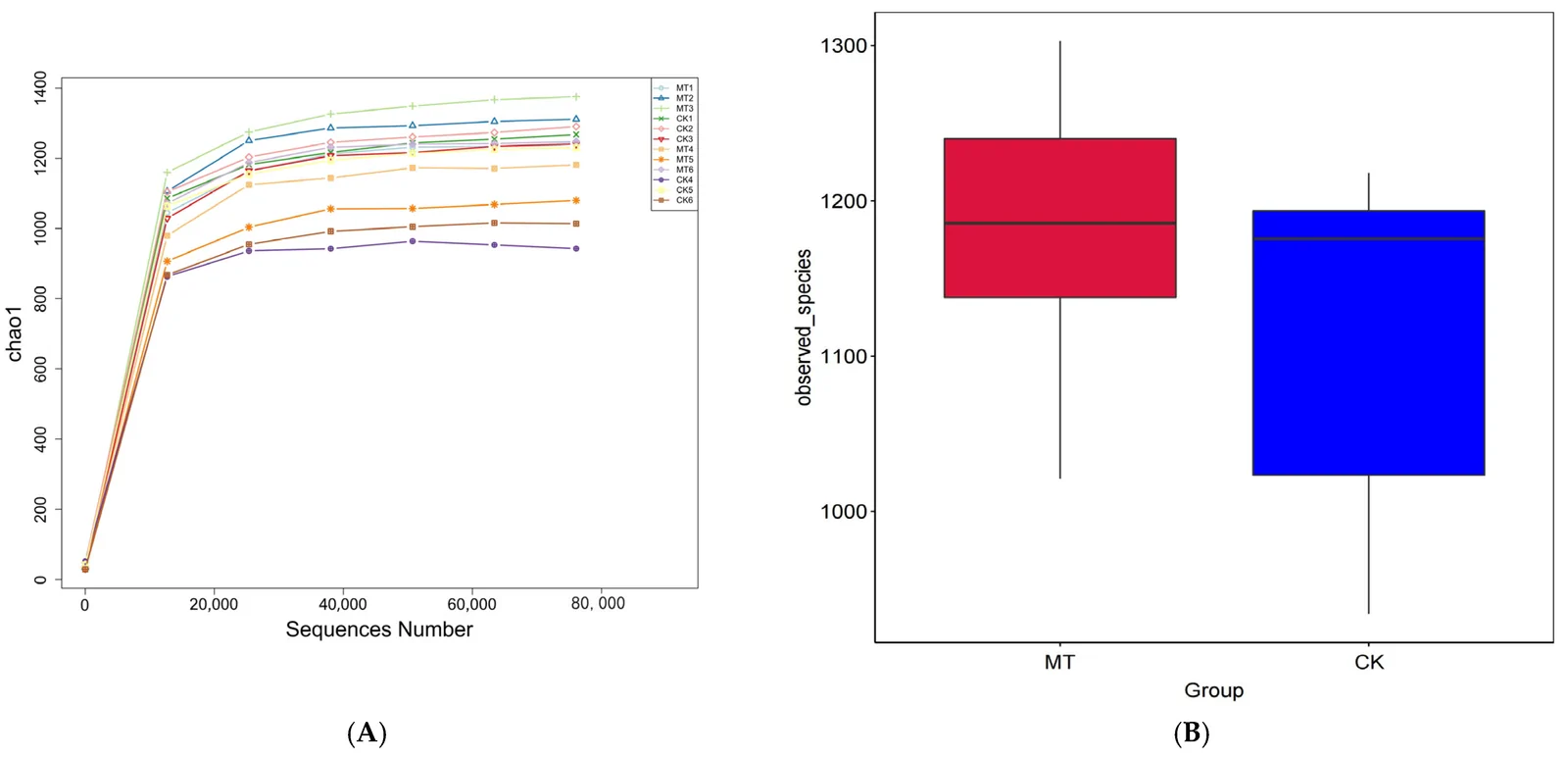
Alpha diversity of rumen bacterial communities in Suffolk ram lambs. (**A**) Rarefaction curves showing the Chao1 index as a function of sequencing depth. (**B**) Box plot of intergroup differences in alpha diversity indices.

As shown in the rarefaction curve (Figure 1A), the Chao1 index curve for each sample gradually increased and then leveled off as sequencing depth increased. When the sequencing depth reached approximately 40,000–60,000 reads, it approached a plateau, indicating that the sequencing data volume was sufficient to cover the vast majority of species in the rumen microbiota. The sequencing depth was reasonable and could accurately reflect the species composition and community structure of the samples.

The results of alpha diversity analysis (Figure 1B) show that the observed species count in the MT group was numerically higher than that in the CK group. However, no significant differences were observed between the two groups in Chao1 (CK: 1163.88, MT: 1239.74, *p* = 0.330), Shannon (CK: 7.55, MT: 7.50, *p* = 0.810), or Simpson (CK: 0.980, MT: 0.978, *p* = 0.740) indices.

#### 3.6.2. Beta Diversity of Rumen Microbiota Structure

**Figure 2:**
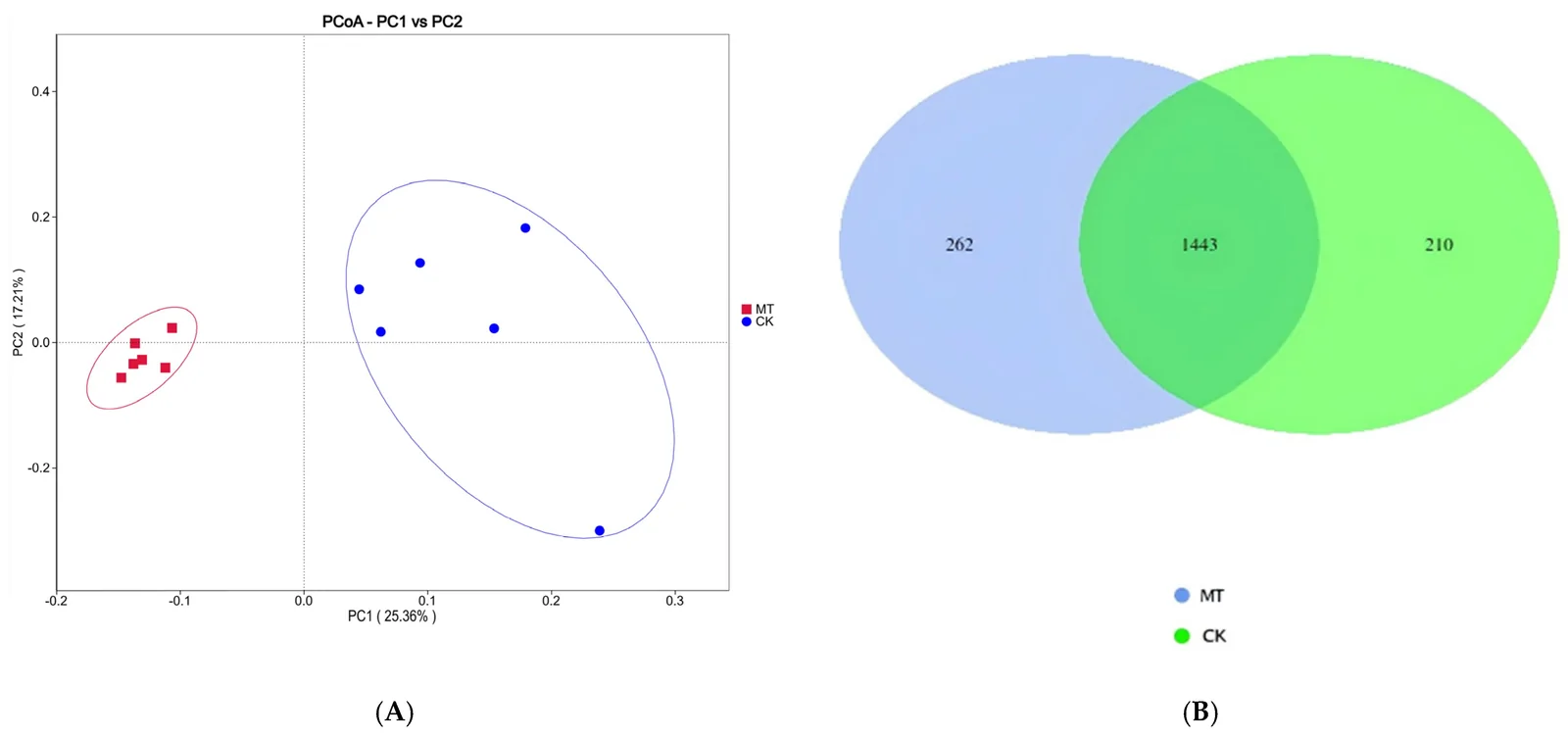
Comparison of rumen microbial community structure between CK and MT groups. (**A**) Principal Coordinate Analysis (PCoA) based on weighted UniFrac distance. (**B**) Venn diagram showing shared and unique OTUs.

The results of Principal Coordinate Analysis (PCoA) based on weighted UniFrac distance (Figure 2A) show that PC1 and PC2 explain 25.36% and 17.21% of the total community variation, respectively. Samples from the MT group and the CK group are clearly separated along the PC1 axis on the PCoA plot and cluster in distinct regions, indicating significant differences in rumen microbiota structure between the two groups. PERMANOVA confirmed a significant difference in community structure between the CK and MT groups (R^2^ = 0.281, *p* = 0.003).

The Venn diagram analysis (Figure 2B) revealed that 1443 operational taxonomic units (OTUs) were shared between the MT and CK groups, constituting the majority of the total OTUs in both groups, suggesting that the rumen microbial communities of the two groups share a common species composition foundation. Additionally, the MT group contained 262 unique OTUs, while the CK group contained 210 unique OTUs, indicating that each group enriched specific microbial taxa.

#### 3.6.3. Analysis of Changes in Rumen Microbial Species Composition

**Figure 3:**
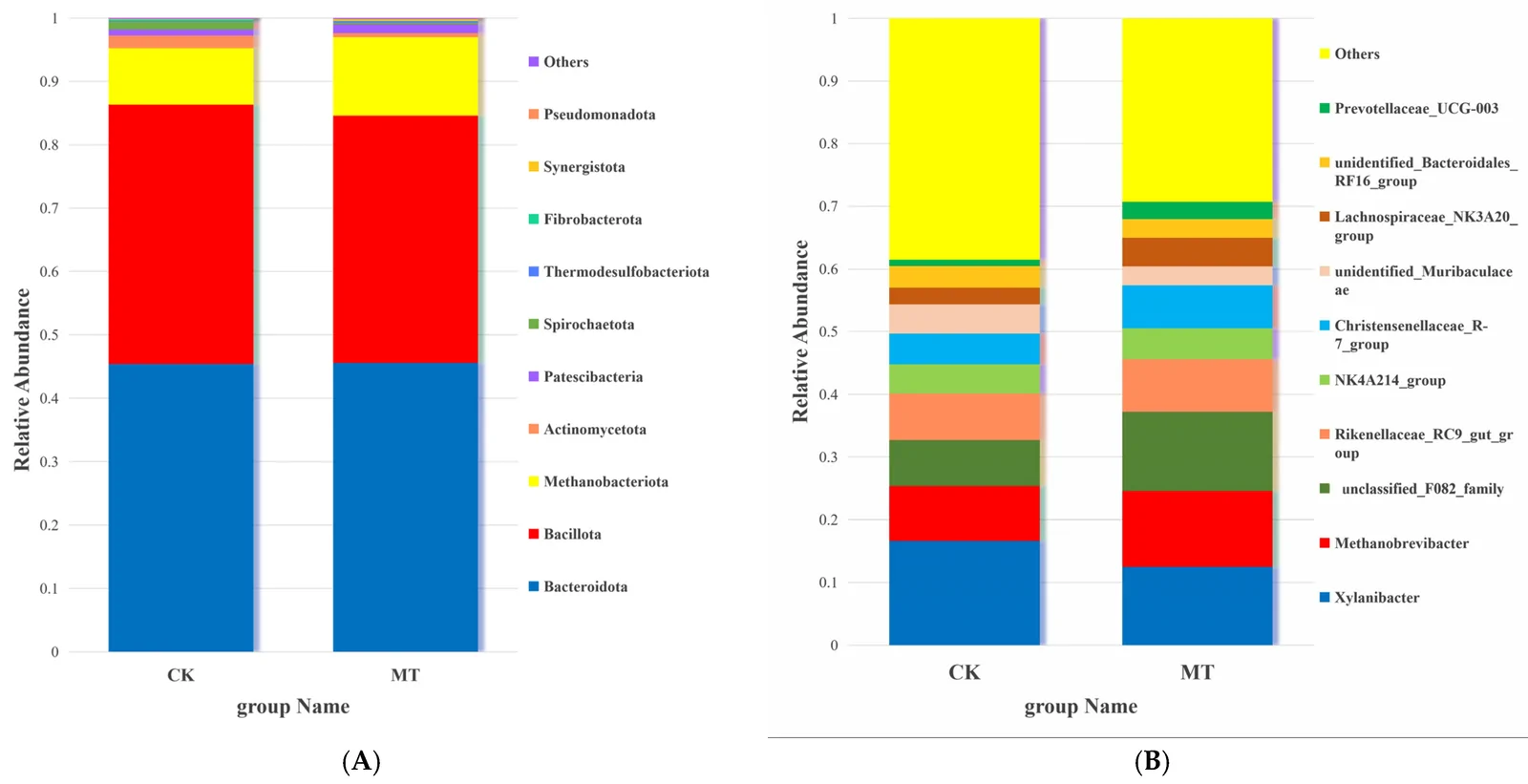
Top 10 species relative abundance bar charts. (**A**) Top 10 bar chart of relative species abundance at the phylum level. (**B**) Top 10 bar chart of relative species abundance at the genus level. The x-axis represents the sample name; the y-axis represents relative abundance. “Others” represents the sum of the relative abundances of all taxa other than the 10 shown in the figure.

As shown in Figure 3A, at the phylum level, the microbial communities of both groups were dominated by *Bacteroidota* and *Bacillota*, which together accounted for more than 84% of the total. The relative abundance of *Spirochaetota* in the CK group was significantly higher than that in the MT group (*p* < 0.01). The relative abundance of the *Synergistota phylum* in the MT group was significantly higher than that in the CK group (*p* < 0.05). No significant differences were observed in the relative abundance of *Fibrobacterota* and *Pseudomonadota* between the two groups (*p* > 0.05).

As shown in Figure 3B, at the genus level, the relative abundance of *unclassified F082 family* in the MT group was significantly higher than that in the CK group (*p* < 0.05). The relative abundance of *Lachnospiraceae_NK3A20_group* in the MT group was significantly higher than that in the CK group (*p* < 0.01). The relative abundance of “Others” in the CK group was significantly higher than that in the MT group (*p* < 0.001). The relative abundances of *Prevotellaceae_UCG-003*, *Christensenellaceae_R-7_group*, and *unidentified_Muribaculaceae* in the MT group showed an increasing trend. No significant differences were observed between the two groups for the remaining genera (*p* > 0.05).

#### 3.6.4. LEfSe Analysis

**Figure 4:**
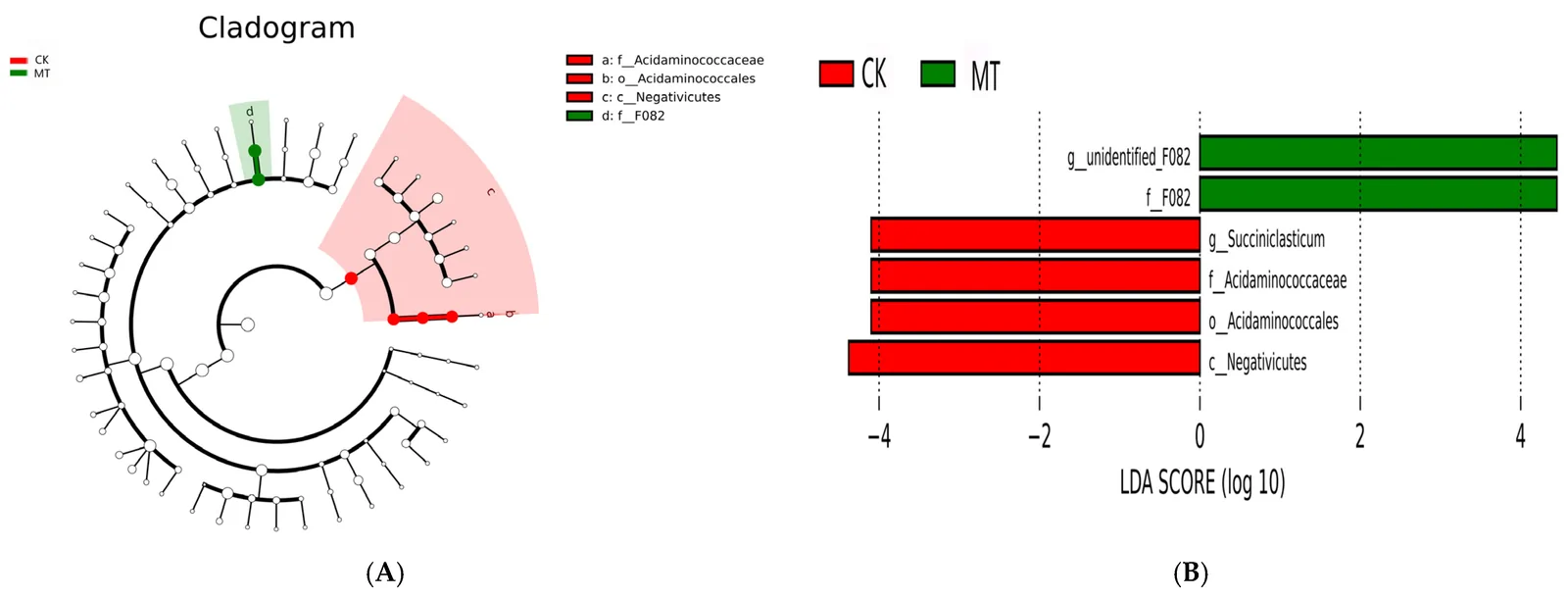
LEfSe analysis of differentially abundant microbial taxa between CK and MT groups. (**A**) Cladogram showing phylogenetic relationships of biomarkers with LDA scores > 4. Circle diameter is proportional to relative abundance. Red nodes indicate taxa enriched in the MT group; green nodes indicate taxa enriched in the CK group. (**B**) LDA score distribution of differentially abundant taxa.

LEfSe analysis was performed to identify specific microbial communities associated with improved production performance. Figure 4A shows the phylogenetic branches with LDA scores > 4. Figure 4B is a bar chart of the LDA score distribution for biomarkers screened between groups. Linear Discriminant Analysis (LDA) results indicate that there are 12 significantly different evolutionary branches between the two groups. At the genus level, the MT group was significantly enriched with the *unclassified F082 family*, which had a higher LDA score. The differential microbial communities enriched in the CK group were primarily concentrated in the Negativicutes class and its subordinate branches.

### 3.7. Correlation Analysis of Rumen Microorganisms with Other Indicators

**Figure 5:**
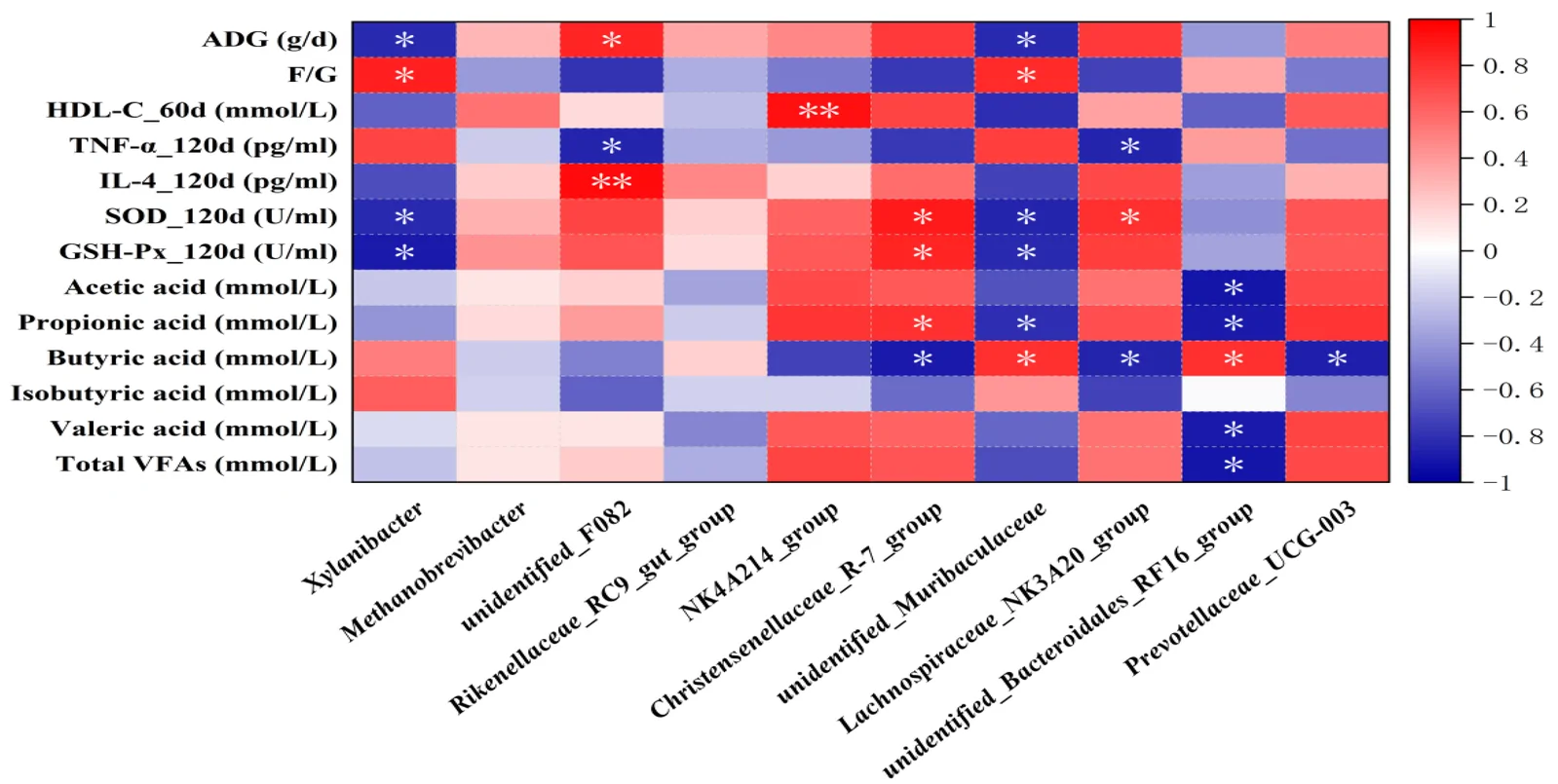
Spearman correlation heatmap between rumen microbial genera and growth performance and physiological indicators in Suffolk ram lambs. Red indicates positive correlation; blue indicates negative correlation. * *p* < 0.05, ** *p* < 0.01. ADG, daily weight gain; F:G, feed-to-gain ratio; TNF-α, tumor necrosis factor-alpha; IL-4, interleukin-4; SOD, superoxide dismutase; GSH-Px, glutathione peroxidase; HDL-C, high-density lipoprotein cholesterol; TVFA, total volatile fatty acids. n = 6 per group for rumen microbiota analysis.

Spearman correlation analysis was performed as an exploratory screen; uncorrected *p*-values are shown. Spearman’s correlation analysis showed (Figure 5) that, regarding growth performance, *Xylanibacter* was significantly negatively correlated with ADG and significantly positively correlated with F:G (*p* < 0.05). *unclassified F082 family* was significantly positively correlated with ADG and significantly negatively correlated with F:G (*p* < 0.05). *Unidentified_Muribaculaceae* showed a significant negative correlation with ADG and a significant positive correlation with F:G (*p* < 0.05).

Regarding immunity and antioxidant activity, *unclassified F082 family* showed a highly significant positive correlation with IL-4 levels on d 120 (*p* < 0.01) and a significant negative correlation with TNF-α levels (*p* < 0.05). *Lachnospiraceae_NK3A20_group* showed a significant negative correlation with TNF-α levels (*p* < 0.05). *unclassified F082 family* showed significant positive correlations with both SOD and GSH-Px activities (*p* < 0.05). *Xylanibacter* and *unidentified_Muribaculaceae* were significantly negatively correlated with both SOD and GSH-Px activities (*p* < 0.05). *unclassified F082 family* was highly significantly positively correlated with HDL-C levels on d 60 (*p* < 0.01).

Regarding VFA, *Christensenellaceae_R-7_group* showed a significant positive correlation with propionic acid content and a significant negative correlation with butyric acid content (*p* < 0.05). *Unidentified_Muribaculaceae* showed a significant positive correlation with butyric acid content (*p* < 0.05). *Unidentified_RF16_group* showed significant negative correlations with butyric acid, valeric acid, and TVFA (*p* < 0.05). *Prevotellaceae_UCG-003* showed significant positive correlations with acetic acid, valeric acid, and TVFA (*p* < 0.05).

## 4. Discussion

### 4.1. Growth Performance

In this study, the MT group exhibited the greatest ADG among all treatments, followed by the ML group, while the MC group showed a diminished response. This substitution-dependent pattern aligns with the findings of Dadashi et al. [8], who reported that silage made from sugar beet pulp and wheat straw had nutritional value comparable to that of corn silage, and Guo et al. [18], who demonstrated that fermented cotton stalk could achieve daily gain close to that of corn silage in fattening sheep. Wang et al. [19] further showed that microbial-enzymatic co-fermentation improved the storage quality and ruminal degradability of cotton stalks, suggesting enhanced fiber utilization in meat sheep. The superior ADG in the MT group likely resulted from the synergistic effect of sugar beet pulp pectin and fermented cotton stalks; pectin provides rapidly fermentable energy, while fermented cotton stalks supply slowly digestible fiber, creating a balanced energy supply curve. The reduced performance in the MC group may be attributable to several factors. First, the MC diet had the highest ADF content (26.5%). Although NDF levels were comparable among groups (Table 1), the increasing ADF proportion suggests a higher lignin-cellulose fraction in MC, potentially reducing fiber digestibility and nutrient availability. Second, the higher inclusion of CSBPS may have negatively affected palatability, thereby limiting dry matter intake. Additionally, the potential accumulation of antinutritional factors (such as gossypol) from cotton stalk-derived materials at the 90% replacement level cannot be excluded, as long-term or high-dose feeding of cotton stalk has been shown to cause free gossypol accumulation and damage to the liver and kidneys in sheep [20].

The increase in ADFI observed in the MT group can be attributed to improved palatability from flavor compounds produced during micro-fermentation, the predominant fermentation of pectin to acetate rather than lactate, which helps stabilize rumen pH and reduces acidosis risk, and increased VFA production which alleviates negative feedback inhibition of dry matter intake [21]. The slightly lower ADFI in the MC group compared to MT likely reflects the effect of high fiber content on rumen fill [22], though the addition of sugar beet pulp still maintained intake above the CK level.

Notably, the magnitude of ADG improvement exceeded that of dry matter intake increase, indicating that improved feed conversion efficiency, rather than increased intake alone, drove the growth response. The reduced F:G ratio in the MT group suggests greater nutrient conversion into body tissue deposition. This may be explained by improved fiber digestibility through enzymatic action during micro-silage treatment [21]; enhanced specific branched-chain VFA production (isobutyrate and valerate) and the tendency to increase propionate production, promoting glucose synthesis and protein deposition; and reduced energy expenditure on non-productive immune and oxidative stress responses [23]. The lack of improvement in the MC group likely reflects that the higher ADF content offset the benefits of micro-silage treatment, prolonging feed retention time and increasing energy loss in feces [24]. Overall, the 60% replacement ratio yielded favorable growth performance, suggesting it is a suitable approach for fattening Suffolk ram lambs under the conditions tested. The growth response observed with CSBPS aligns with recent findings on other agricultural by-products. İnanç and Arık [25] reported that dried white mulberry pomace supplementation at 150 g/d increased live weight gain by 60.9% in Merino lambs compared with 46.7% in controls, although the between-group differences did not reach statistical significance. In contrast, the 60% CSBPS replacement in the present study achieved a statistically significant 38.4% improvement in daily weight gain and a 22.1% reduction in feed-to-gain ratio, suggesting that the cotton stalk–sugar beet pulp combination may offer more consistent and pronounced performance benefits under intensive fattening conditions.

### 4.2. Serum Biochemical Indicators

The stability of TP, ALB, TC, TG, and BUN across all treatment groups indicates that replacing whole-plant corn silage with CSBPS did not adversely affect basal nutritional metabolism [24]. TP and ALB remained within normal physiological ranges. The lack of intergroup differences in BUN further supports that nitrogen metabolic balance was maintained despite changes in roughage source [26]. The overall stability of these serum biochemical indicators, together with the absence of disturbances in energy-related metabolic pathways [27], confirms that replacing up to 60% of the diet with CSBPS is safe and feasible.

The elevated GLB in the MC group on d 60 may reflect immune system activation by the high proportion of micro-silage substitution, consistent with the increased IgA and IgG levels observed. The higher HDL-C in MT and MC groups suggests enhanced hepatic lipoprotein synthesis and secretion, which benefits cholesterol reverse transport and lipid metabolism. The transient reduction in blood glucose in the ML group on d 60 may reflect stabilization of carbohydrate intake and reduced glycemic fluctuation. In ruminants, blood glucose is primarily maintained via gluconeogenesis, and increased propionate production can promote hepatic gluconeogenesis [22]; however, propionate also stimulates insulin secretion, and improved insulin sensitivity may accelerate peripheral glucose utilization [28]. The normalization of glucose levels by d 120 suggests that the animals established a new metabolic balance through endocrine regulation.

### 4.3. Serum Immunological Parameters

The elevated serum IgA, IgG, and IgM levels in MT and MC groups, along with reduced IL-1β and increased IL-4, indicate enhanced humoral immunity and suppressed inflammation. Notably, TNF-α levels in all groups showed a progressive decline over the course of the experiment, which likely reflects the physiological maturation and adaptation of the growing lambs to the housing and feeding conditions. Given the significant baseline imbalance at d 0, ANCOVA was applied to adjust for the elevated initial TNF-α in the CK group. After this adjustment, TNF-α still exhibited a significant linear decrease with increasing CSBPS substitution across d 30–120, and the CSBPS-supplemented groups consistently exhibited lower estimated marginal means than the control group. This pattern suggests that CSBPS supplementation was associated with reduced TNF-α levels; however, due to the baseline imbalance, this finding should be interpreted as supportive evidence of a potential anti-inflammatory effect rather than a definitive causal relationship. TNF-α and IL-1β are central mediators of the inflammatory response; their reduction suggests lower inflammatory stress, allowing more protein and energy to be allocated toward muscle tissue synthesis [23].

Several mechanisms may explain the immunomodulatory effects of CSBPS. Lactic acid bacteria and their metabolites (e.g., extracellular polysaccharides and organic acids) produced during fermentation possess immunomodulatory activity, promoting gut-associated lymphoid tissue (GALT) development and antibody secretion [29]. Soluble fibers such as pectin, abundant in sugar beet pulp, ferment in the rumen to produce SCFAs, particularly butyrate, which can regulate immune cell function by activating the GPR43 receptor and inhibiting histone deacetylases (HDACs), promoting IL-4 secretion while suppressing TNF-α production [30,31]. Additionally, micro-silage treatment may degrade antinutritional factors in cotton stalks, reducing their irritation to immune organs. The shift toward a Th2-type anti-inflammatory immune response, indicated by the contrasting increase in IL-4 and decrease in TNF-α, is consistent with improved intestinal mucosal immune homeostasis. The reduction in inflammatory stress likely conserved energy and protein otherwise expended on immune responses, redirecting these nutrients toward tissue deposition [23], consistent with the improved feed conversion efficiency in the MT group.

### 4.4. Serum Antioxidant Parameters

The increased SOD and GSH-Px activities across all treatment groups indicate enhanced antioxidant defense capacity. The enhanced antioxidant capacity can be attributed to several factors: sugar beet pulp is rich in methyl donors such as betaine, which can promote antioxidant enzyme gene expression through one-carbon metabolism and osmotic regulation [32]; phenolic compounds and antioxidant peptides produced during micro-silage fermentation can directly scavenge free radicals [33]; and SCFAs (particularly butyrate) produced by rumen microbial fermentation can activate the Nrf2 signaling pathway, promoting SOD and GSH-Px synthesis [34]. For MDA, a reduction was observed only in the 90% replacement group at d 90, with no significant differences among groups at d 120, suggesting that the effect of CSBPS on lipid peroxidation may be transient or limited under the current experimental conditions. The temporal fluctuation in MDA measurements, particularly the decline in the control group from d 90 to d 120, may reflect normal biological variation or assay variability [35,36]. This enhanced antioxidant capacity likely protected the mucosal barrier function of the digestive tract, promoting efficient nutrient absorption and contributing to the reduced F:G ratio observed in the MT group. Furthermore, oxidative stress is closely linked to inflammation and ROS can activate NF-κB signaling to promote pro-inflammatory factor production, whereas enhanced antioxidant enzyme activity can block this pathway [34]. This is corroborated by the reduced TNF-α levels discussed above, suggesting that enhanced antioxidant and anti-inflammatory states synergistically support improved growth performance.

### 4.5. Rumen Fermentation Parameters

The absence of significant differences in rumen pH, acetate, propionate, butyrate, or TVFA between MT and CK groups suggests that the 60% CSBPS replacement did not disrupt ruminal fermentation stability, but rather optimized specific metabolite production. Rumen pH was maintained within the normal physiological range (6.18–6.34) [24], confirming that the lactic acid-free fermentation characteristic of sugar beet pulp pectin effectively stabilized the rumen environment, consistent with the increased dry matter intake observed.

The significantly higher isobutyrate and valerate levels in the MT group are noteworthy. These branched-chain volatile fatty acids (BCVFAs) originate from microbial deamination of branched-chain amino acids and serve as important growth factors for fiber-degrading and protein-degrading bacteria [37]. BCVFAs can provide carbon skeletons and energy sources for rumen microorganisms, promoting proliferation of fiber-degrading bacteria such as the *Lachnospiraceae NK3A20 group* [38], thereby improving cellulose and hemicellulose digestibility. The elevated BCVFA levels in the MT group are consistent with the enrichment of fiber-degrading bacteria observed in this study, forming a positive feedback loop: fermentation products promote microbial proliferation, which in turn enhances fiber degradation and energy release. This mechanism likely explains the improved feed conversion efficiency in the MT group.

### 4.6. Rumen Microbiome

While alpha diversity did not differ between groups, PCoA analysis revealed distinct microbial community structures, indicating that CSBPS replacement reshaped the microbiota rather than simply altering diversity. The higher number of unique OTUs in the MT group suggests enrichment of specific functional microbial communities.

At the phylum level, the dominance of *Bacteroidota* and *Bacillota* is consistent with the fundamental characteristics of the rumen microbiota in ruminants [39]. The reduced *Spirochaetota* abundance in the MT group reflects improved feed quality, as Liu et al. [40] demonstrated that Treponema (a genus within Spirochaetota) was more abundant in the rumen microbiota colonizing rice straw compared with alfalfa hay in ruminants. The increased Synergistota abundance in the MT group, representing amino acid-degrading bacteria involved in protein hydrolysis and BCVFA production, directly corresponds to the elevated isobutyrate and valerate levels observed.

At the genus level, the enrichment of *Lachnospiraceae_NK3A20_group* and the *F082 family* in the MT group is notable. Lachnospiraceae are core rumen microbiota involved in cellulose and hemicellulose degradation, producing abundant SCFAs (particularly butyrate) and playing a key role in maintaining rumen epithelial health and promoting host energy acquisition [41]. *Lachnospiraceae-related* taxa are significantly correlated with NDF and ADF degradation rates in sheep rumen, serving as important microbial biomarkers of feed conversion efficiency. The enrichment of these fiber-degrading bacteria directly explains the improved feed efficiency in the MT group. The *F082 family*, another recently reported uncultured bacterial group associated with fiber degradation and VFA production, was also enriched in MT [41], further supporting the enhancing effect of micro-silage on fiber degradation. These microbial shifts align with previous investigations on the effects of different proportions of beet pulp and cotton stalk fermentation on nutrient components and rumen fermentation parameters.

### 4.7. Correlation Analysis and Integrated Mechanism

Spearman’s correlation analysis provided statistical evidence linking specific microbial taxa to host physiological responses. The positive correlation between *unclassified F082 family* and ADG, along with its negative correlation with F:G, confirms the key role of this bacterial group in promoting growth and feed efficiency. *Conversely*, the negative correlation of *Xylanibacter* with ADG and positive correlation with F:G suggests this taxon may be associated with less favorable fiber degradation or fermentation patterns.

The positive correlations between fiber-degrading bacteria (*unclassified F082 family* and *Lachnospiraceae_NK3A20_group*) and anti-inflammatory (IL-4) and antioxidant (SOD, GSH-Px) indicators, along with their negative correlations with TNF-α, indicate that enrichment of these bacteria is closely associated with improved immune and antioxidant status. The likely mechanism is that SCFAs (particularly butyrate) produced by fiber-degrading bacteria enter the circulatory system and regulate peripheral immune cell function via GPR43 activation and HDAC inhibition, reducing pro-inflammatory factor secretion [30,31]. Additionally, the enrichment of fiber-degrading bacteria may indirectly enhance systemic antioxidant capacity by reducing intestinal oxidative stress and strengthening mucosal barrier function [34].

The differential correlations of *Christensenellaceae_R-7_group* (positively correlated with propionate, negatively with butyrate), *unidentified_Muribaculaceae* (positively correlated with butyrate), and *Prevotellaceae_UCG-003* (positively correlated with acetate, valerate, and TVFA) reveal functional differentiation among microbial communities in VFA production. These correlations support a comprehensive model: 60% CSBPS replacement enriches fiber-degrading bacteria (*Lachnospiraceae and F082 family*), which improves fiber digestibility and enhances specific BCVFA production, increasing dry matter intake and enhancing feed conversion efficiency, ultimately boosting daily weight gain. Concurrently, SCFAs such as butyrate enter the circulatory system, promoting anti-inflammatory responses and enhancing antioxidant enzyme activity. This alleviated immune and oxidative stress, reducing maintenance energy expenditure and allowing more nutrients to be utilized for weight gain.

#### Limitations

Several limitations of this study should be acknowledged. First, the experimental unit was the pen (n = 3 per treatment), which may limit statistical power for detecting subtle treatment effects. Second, the 16S rRNA gene sequencing and rumen fermentation analyses were performed only on the CK and MT groups, representing a post hoc selection based on growth and serum performance results; therefore, these findings should be considered descriptive and exploratory rather than confirmatory. Third, free and total gossypol concentrations in the CSBPS and TMRs were not measured, and detailed fermentation quality parameters (pH, organic acids, ammonia-N) of the CSBPS were not determined. Fourth, total tract digestibility was not determined; therefore, claims regarding fiber digestion should be interpreted with caution. Fifth, the elevated baseline TNF-α in the CK group at d 0, while adjusted for by ANCOVA, may still complicate the interpretation of treatment effects on this acute-phase marker. Sixth, given the large number of serum variables and time points measured, the immunological and antioxidant findings should be considered exploratory, and no formal correction for multiplicity was applied. Finally, the experiment was conducted only in Suffolk ram lambs, and whether the findings apply to other breeds, sexes, or physiological stages requires further investigation.

## 5. Conclusions

This study evaluated the effects of replacing whole-plant corn silage with cotton stalk-sugar beet pulp silage at 0%, 30%, 60%, and 90% on growth performance, serum immunity and antioxidant status, rumen fermentation, and microbiota in Suffolk ram lambs. The 60% replacement ratio (MT) yielded favorable performance, increasing average daily gain by 38.37% compared with the control and decreasing feed-to-gain ratio by 22.13%. The MT and MC groups showed improved immunoglobulin levels, reduced pro-inflammatory cytokines, and enhanced antioxidant enzyme activity. Rumen isobutyrate and valerate levels increased, and fiber-degrading bacteria (*Lachnospiraceae_NK3A20_group*) were enriched in the MT group. In summary, under the conditions tested, replacing 60% of whole-plant corn silage with cotton stalk-sugar beet pulp silage proved to be a suitable approach for fattening Suffolk ram lambs, as it optimizes rumen fermentation and microbiota, improves immune and antioxidant status, and enhances performance. This study provides a basis for efficient utilization of agricultural by-products in meat sheep production.

## Figures and Tables

**Table 1:** Diet formulation and nutritional levels.

Item	CK ^1^	ML30 ^1^	MT60 ^1^	MC90 ^1^
Ingredients % ^2^				
Whole-plant corn silage	21.00	15.00	8.00	2.00
cotton stalk and sugar beet pulp mixed silage	0.00	5.00	11.00	16.00
Alfalfa hay	6.00	6.00	6.00	6.00
Corn stover	13.00	13.00	13.00	13.00
Corn grain	31.00	32.00	33.00	33.50
Cottonseed meal	6.00	6.00	6.00	6.00
Soybean meal	9.00	9.00	9.00	9.50
Wheat bran	6.00	6.00	6.00	6.00
Premix ^3^	3.00	3.00	3.00	3.00
Fermented wet material ^4^	3.00	3.00	3.00	3.00
Sodium bicarbonate	1.00	1.00	1.00	1.00
Salt	1.00	1.00	1.00	1.00
Total	100.00	100.00	100.00	100.00
Nutrient levels ^5^				
Crude protein, %	13.68	13.72	13.93	13.81
Ether extract, %	4.76	4.54	4.34	4.17
Metabolizable energy, MJ/kg	8.10	8.23	8.37	8.50
Crude ash, %	8.18	8.75	8.86	8.12
Neutral detergent fiber, %	36.33	37.74	36.45	36.42
Acid detergent fiber, %	23.5	24.5	25.5	26.5
Calcium, %	0.73	0.72	0.76	0.79
Total phosphorus, %	0.38	0.38	0.38	0.38

^1^ CK group: Control group, in which whole-plant corn silage in the basal diet was replaced with 0% cotton stalks and sugar beet pulp mixed silage; ML group: Whole-plant corn silage in the basal diet was replaced with 30% cotton stalks and sugar beet pulp mixed silage; MT group: Replaced whole-plant corn silage in the basal diet with 60% cotton stalks and sugar beet pulp mixed silage; MC group: Replaced whole-plant corn silage in the basal diet with 90% cotton stalks and sugar beet pulp mixed silage. Replacement ratios (30%, 60%, 90%) indicate the proportion of the control-level corn silage (21.00%) that was substituted by CSBPS. The actual as-fed inclusions of CSBPS (5.00%, 11.00%, 16.00%) are independent formulation values determined by nutrient re-balancing and do not represent a 1:1 fresh-weight substitution. ^2^ All roughage used in the trial was uniformly provided by the experimental site, and all concentrate feed was purchased from Xinjiang Tiankang Feed Co., Ltd. (Urumqi, Xinjiang, China). ^3^ The premix formulation was as follows: Vitamin A 130,000–250,000 IU, Vitamin D3 40,000–100,000 IU, Vitamin E ≥ 1400 IU, Copper (basic copper chloride) 550–800 mg, Iron (ferrous sulfate) 1500–7000 mg, Manganese (manganese sulfate) 1100–3000 mg, zinc (zinc sulfate) 800–2000 mg, Iodine (calcium iodate) 20–30 mg, selenium 8–12 mg, calcium 10–20%, total phosphorus 1.5%, sodium chloride 12–20%, moisture ≤10.0%. ^4^ Fermented Wet Feed: A feed produced by fermenting flaked corn, cottonseed meal, and wheat bran using a mixed culture of *Lactobacillus acidophilus*, *Saccharomyces cerevisiae*, and *Bacillus licheniformis*. ^5^ Nutritional levels are actual measured values; metabolizable energy is a calculated value. Values are presented on a dry matter basis. Each value represents the mean of three analytical replicates (*n* = 3).

**Table 2:** Chemical composition of CSBPS and whole-plant corn silage (DM basis).

Item	CSBPS	Whole-Plant Corn Silage
Dry matter, %	33.36	33.41
Crude protein, %	5.31	8.52
Ether extract, %	1.98	5.01
Crude ash, %	6.65	7.14
Neutral detergent fiber, %	64.48	42.01
Acid detergent fiber, %	40.24	28.31
Gross Energy, MJ/kg	14.33	13.51

CSBPS, cotton stalk and sugar beet pulp mixed silage. Values are presented on a dry matter basis. All values are means of three independent silos (n = 3).

**Table 3:** Effects of different replacement ratios of cotton stalks and sugar beet pulp in micro-silage on the growth performance of Suffolk ram lambs.

Indicator	Group				*p*-Value
	CK	ML	MT	MC	
Initial weight, kg	32.33 ± 1.57	32.22 ± 1.01	32.47 ± 1.55	32.42 ± 2.35	0.999
Final weight, kg	54.09 ± 1.71 ^C^	61.13 ± 2.97 ^A^	62.58 ± 2.81 ^A^	56.96 ± 3.12 ^B^	0.001
Net weight gain, kg	21.76 ± 2.04 ^C^	28.91 ± 2.73 ^A^	30.11 ± 2.06 ^A^	24.54 ± 3.84 ^B^	0.001
Average daily gain, g/d	181.32 ± 17.01 ^C^	240.90 ± 22.79 ^A^	250.93 ± 17.21 ^A^	204.50 ± 32.01 ^B^	0.001
Average daily dry matter intake, kg/d	1.52 ± 0.01 ^C^	1.59 ± 0.02 ^B^	1.65 ± 0.02 ^A^	1.63 ± 0.01 ^AB^	0.002
Feed-to-gain ratio	8.45 ± 0.82 ^A^	6.66 ± 0.66 ^B^	6.58 ± 0.41 ^B^	8.13 ± 1.17 ^A^	0.001

^A–C^ Different uppercase letters in the same row indicate highly significant differences (*p* < 0.01); identical letters indicate no significant difference. CK: control group; ML: 30% replacement; MT: 60% replacement; MC: 90% replacement. Values are presented as mean ± SEM of pen means (n = 3 pens per treatment, 7 lambs per pen).

**Table 4:** Effects of different replacement ratios of cotton stalks and sugar beet pulp in micro-silage on serum biochemical indicators in Suffolk ram lambs.

Parameter	Time	Group				*p*-Value
		CK	ML	MT	MC	
Total protein, g/L	0 d	63.75 ± 2.73	67.66 ± 8.41	65.82 ± 6.08	64.41 ± 7.51	0.887
	30 d	66.18 ± 3.51	72.41 ± 5.17	70.27 ± 3.43	71.31 ± 2.07	0.262
	60 d	63.58 ± 3.78	64.73 ± 4.52	68.53 ± 2.72	69.75 ± 0.63	0.137
	90 d	65.46 ± 6.36	74.60 ± 9.04	68.76 ± 5.18	69.22 ± 3.16	0.411
	120 d	70.21 ± 6.19	71.61 ± 1.11	66.20 ± 1.08	71.11 ± 3.06	0.300
Albumin, g/L	0 d	26.99 ± 3.11	26.95 ± 2.62	26.89 ± 1.98	29.61 ± 3.75	0.622
	30 d	26.86 ± 2.13	27.16 ± 4.04	27.97 ± 2.54	25.66 ± 3.56	0.843
	60 d	28.21 ± 0.68	27.91 ± 1.95	31.08 ± 1.11	27.20 ± 3.22	0.165
	90 d	32.12 ± 0.69	31.05 ± 4.56	33.35 ± 0.91	30.64 ± 0.57	0.535
	120 d	31.79 ± 3.93	33.69 ± 3.11	30.91 ± 0.81	34.36 ± 0.23	0.369
Globulin, g/L	0 d	36.76 ± 2.21	40.71 ± 5.78	38.93 ± 7.21	34.80 ± 3.95	0.553
	30 d	39.32 ± 4.89	45.25 ± 8.31	42.30 ± 3.99	45.65 ± 2.61	0.483
	60 d	35.37 ± 4.35 ^b^	36.83 ± 3.02 ^ab^	37.45 ± 3.50 ^ab^	42.55 ± 3.03 ^a^	0.047
	90 d	33.35 ± 6.81	43.55 ± 13.61	35.41 ± 4.56	38.58 ± 2.71	0.477
	120 d	38.41 ± 4.24	37.92 ± 3.86	35.29 ± 0.58	36.75 ± 3.14	0.670
Total cholesterol, mmol/L	0 d	1.16 ± 0.09	1.16 ± 0.38	1.10 ± 0.09	1.22 ± 0.19	0.659
	30 d	1.12 ± 0.16	1.13 ± 0.07	1.13 ± 0.18	1.14 ± 0.26	0.999
	60 d	1.02 ± 0.21	1.23 ± 0.41	1.23 ± 0.08	1.16 ± 0.09	0.686
	90 d	1.19 ± 0.17	1.26 ± 0.06	1.35 ± 0.23	1.38 ± 0.13	0.506
	120 d	1.57 ± 0.24	1.29 ± 0.28	1.43 ± 0.02	1.42 ± 0.13	0.457
Triglycerides, mmol/L	0 d	0.23 ± 0.07	0.32 ± 0.15	0.20 ± 0.03	0.18 ± 0.04	0.291
	30 d	0.19 ± 0.07	0.22 ± 0.05	0.19 ± 0.02	0.21 ± 0.04	0.832
	60 d	0.31 ± 0.16	0.22 ± 0.03	0.33 ± 0.05	0.28 ± 0.01	0.493
	90 d	0.33 ± 0.13	0.30 ± 0.17	0.46 ± 0.12	0.37 ± 0.04	0.452
	120 d	0.24 ± 0.13	0.22 ± 0.03	0.17 ± 0.08	0.24 ± 0.05	0.729
High-density lipoprotein cholesterol, mmol/L	0 d	0.83 ± 0.09	0.85 ± 0.21	0.78 ± 0.03	0.97 ± 0.06	0.303
	30 d	0.85 ± 0.11	0.90 ± 0.09	0.86 ± 0.09	0.91 ± 0.06	0.760
	60 d	0.78 ± 0.14 ^ab^	0.63 ± 0.15 ^b^	0.89 ± 0.07 ^a^	0.89 ± 0.05 ^a^	0.045
	90 d	0.82 ± 0.12	0.84 ± 0.15	0.91 ± 0.18	0.94 ± 0.03	0.654
	120 d	0.89 ± 0.18	0.86 ± 0.22	0.89 ± 0.07	1.03 ± 0.12	0.595
LDL-C, mmol/L	0 d	0.38 ± 0.05	0.37 ± 0.17	0.30 ± 0.05	0.38 ± 0.09	0.761
	30 d	0.34 ± 0.06	0.33 ± 0.06	0.35 ± 0.08	0.34 ± 0.14	0.993
	60 d	0.32 ± 0.10	0.49 ± 0.21	0.39 ± 0.04	0.36 ± 0.06	0.393
	90 d	0.39 ± 0.06	0.43 ± 0.05	0.47 ± 0.08	0.47 ± 0.09	0.596
	120 d	0.60 ± 0.08	0.44 ± 0.16	0.51 ± 0.02	0.45 ± 0.06	0.259
Creatinine, μmol/L	0 d	112.24 ± 5.09	104.67 ± 34.26	107.26 ± 12.33	112.65 ± 13.61	0.947
	30 d	102.07 ± 7.41	99.92 ± 9.21	101.41 ± 6.95	88.83 ± 11.79	0.307
	60 d	97.41 ± 1.97	107.64 ± 20.17	104.13 ± 7.53	96.14 ± 17.67	0.717
	90 d	99.07 ± 5.83	105.61 ± 15.03	95.95 ± 15.72	84.73 ± 10.49	0.292
	120 d	107.81 ± 7.55 ^a^	93.40 ± 3.95 ^b^	95.68 ± 9.16 ^ab^	96.48 ± 6.71 ^ab^	0.036
Urea nitrogen, mg/dL	0 d	24.93 ± 3.85	24.61 ± 5.44	25.36 ± 1.85	24.09 ± 3.01	0.979
	30 d	14.62 ± 3.29	16.97 ± 1.21	15.78 ± 0.38	17.24 ± 1.75	0.398
	60 d	18.26 ± 2.56	21.25 ± 1.44	19.71 ± 3.15	16.82 ± 1.78	0.194
	90 d	17.85 ± 1.38	18.64 ± 4.24	17.77 ± 2.05	16.91 ± 4.32	0.932
	120 d	22.80 ± 0.44	22.49 ± 0.18	19.31 ± 4.22	20.45 ± 3.37	0.39
Glucose, mmol/L	0 d	4.01 ± 0.08	3.36 ± 0.16	3.59 ± 0.48	3.58 ± 0.59	0.298
	30 d	3.38 ± 0.77	3.24 ± 0.20	3.52 ± 0.26	3.25 ± 0.03	0.826
	60 d	4.28 ± 0.64 ^a^	3.03 ± 0.85 ^b^	3.38 ± 0.21 ^ab^	3.44 ± 0.22 ^ab^	0.047
	90 d	4.64 ± 0.48	4.15 ± 0.65	4.71 ± 0.25	4.72 ± 0.40	0.442
	120 d	4.17 ± 0.28	3.92 ± 0.41	4.14 ± 0.15	3.95 ± 0.31	0.683

^a,b^ Different lowercase letters in the same row indicate significant differences (*p* < 0.05). Identical letters indicate no significant difference. CK: control group; ML: 30% replacement; MT: 60% replacement; MC: 90% replacement. Values are presented as mean ± SEM of pen means (n = 3 pens per treatment, 7 lambs per pen).

**Table 5:** Effects of different replacement ratios of cotton stalks and sugar beet pulp in micro-silage on serum immune parameters in Suffolk ram lambs.

Item	Time	Group				*p*-Value
		CK	ML	MT	MC	
Immunoglobulin A, g/L	0 d	0.99 ± 0.06	1.03 ± 0.11	1.01 ± 0.04	0.96 ± 0.07	0.645
	30 d	0.94 ± 0.06 ^B^	1.02 ± 0.09 ^B^	1.13 ± 0.04 ^A^	1.14 ± 0.02 ^A^	0.008
	60 d	1.10 ± 0.17	1.13 ± 0.04	1.18 ± 0.04	1.23 ± 0.05	0.402
	90 d	0.99 ± 0.08 ^b^	1.05 ± 0.09 ^b^	1.18 ± 0.02 ^a^	1.17 ± 0.04 ^a^	0.018
	120 d	1.14 ± 0.19	1.19 ± 0.03	1.23 ± 0.04	1.27 ± 0.08	0.521
Immunoglobulin G, g/L	0 d	16.52 ± 2.91	14.97 ± 4.15	16.06 ± 4.65	18.11 ± 2.37	0.767
	30 d	16.04 ± 0.82 ^C^	17.05 ± 0.24 ^B^	17.82 ± 0.47 ^AB^	18.54 ± 0.17 ^A^	0.001
	60 d	16.81 ± 0.72 ^b^	18.14 ± 1.03 ^ab^	18.28 ± 0.37 ^ab^	19.08 ± 0.77^a^	0.037
	90 d	17.61 ± 0.12 ^b^	18.26 ± 1.13 ^b^	19.02 ± 0.81 ^ab^	20.46 ± 0.73 ^a^	0.011
	120 d	17.97 ± 0.42 ^C^	18.69 ± 0.85 ^BC^	20.10 ± 0.88 ^AB^	20.69 ± 0.88 ^A^	0.001
Immunoglobulin M, g/L	0 d	0.59 ± 0.06	0.58 ± 0.03	0.55 ± 0.18	0.64 ± 0.02	0.717
	30 d	0.55 ± 0.02 ^b^	0.59 ± 0.07 ^ab^	0.64 ± 0.04 ^ab^	0.64 ± 0.03 ^a^	0.049
	60 d	0.58 ± 0.02	0.57 ± 0.09	0.62 ± 0.09	0.66 ± 0.02	0.327
	90 d	0.61 ± 0.03 ^B^	0.61 ± 0.06 ^AB^	0.67 ± 0.02 ^AB^	0.69 ± 0.05 ^A^	0.007
	120 d	0.63 ± 0.06 ^B^	0.68 ± 0.03 ^AB^	0.69 ± 0.03 ^AB^	0.73 ± 0.02 ^A^	0.008
Tumor Necrosis Factor-α, pg/mL	0 d	86.01 ± 4.52 ^A^	52.07 ± 3.13 ^C^	55.18±9.04 ^BC^	65.22±4.35 ^B^	0.001
	30 d	59.84 ± 1.62 ^A^	56.22 ± 1.08 ^B^	53.40 ± 0.92 ^C^	49.94 ± 0.65 ^D^	0.001
	60 d	60.61 ± 3.39 ^A^	52.96 ± 2.27 ^AB^	51.84 ± 1.93 ^BC^	49.20 ± 1.36 ^C^	0.008
	90 d	61.65 ± 3.67 ^A^	47.58 ± 2.46 ^B^	46.06 ± 2.09 ^B^	45.59 ± 1.47 ^B^	0.006
	120 d	53.83 ± 3.53 ^A^	48.20 ± 2.36 ^B^	46.11 ± 2.01 ^B^	43.92 ± 1.41 ^C^	0.003
Interleukin-1β, pg/mL	0 d	22.94 ± 2.64	21.81 ± 0.47	24.01 ± 2.66	20.09 ± 2.57	0.261
	30 d	21.83 ± 0.91 ^a^	22.25 ± 0.34 ^a^	21.02 ± 1.28 ^ab^	19.81 ± 1.11 ^b^	0.046
	60 d	21.50 ± 1.28	20.79 ± 0.89	20.09 ± 1.35	18.78 ± 1.82	0.173
	90 d	20.18 ± 1.11	19.36 ± 1.31	18.17 ± 0.35	17.35 ± 2.73	0.225
	120 d	19.54 ± 0.48 ^A^	17.71 ± 0.85 ^B^	15.58 ± 0.61 ^C^	15.04 ± 0.56 ^C^	0.001
Interleukin-4, pg/mL	0 d	4.41 ± 0.64	5.10 ± 1.03	5.40 ± 0.57	4.24 ± 1.46	0.461
	30 d	4.39 ± 0.18	4.51 ± 0.41	5.06 ± 0.58	5.18 ± 0.51	0.146
	60 d	4.69 ± 0.09 ^B^	4.81± 0.38 ^B^	5.44 ± 0.46 ^A^	5.82 ± 0.06 ^A^	0.005
	90 d	4.77 ± 0.15 ^C^	5.03 ± 0.28 ^C^	5.61 ± 0.34 ^B^	6.31 ± 0.15 ^A^	0.001
	120 d	4.98 ± 0.09 ^C^	5.29 ± 0.26 ^C^	5.88 ± 0.44 ^B^	6.63 ± 0.24 ^A^	0.001

^A–D^ Different uppercase letters in the same row indicate highly significant differences (*p* < 0.01); ^a,b^ different lowercase letters indicate significant differences (*p* < 0.05). Identical letters indicate no significant difference. CK: control group; ML: 30% replacement; MT: 60% replacement; MC: 90% replacement. Values are presented as mean ± SEM of pen means (n = 3 pens per treatment, 7 lambs per pen).

**Table 6:** Effects of different replacement ratios of cotton stalks and sugar beet pulp in micro-silage on serum antioxidant parameters in Suffolk ram lambs.

Item	Time	Group				*p*-Value
		CK	ML	MT	MC	
Superoxide dismutase, U/mL	0 d	66.25 ± 2.22	67.01 ± 0.51	66.12 ± 2.06	66.12 ± 2.11	0.922
	30 d	62.83 ± 1.49 ^C^	66.81 ± 0.7 ^B^	70.71 ± 2.11 ^A^	72.44 ± 1.12 ^A^	0.001
	60 d	65.08 ± 4.38 ^b^	71.09 ± 0.42 ^a^	73.72 ± 4.33 ^a^	76.02 ± 0.24 ^a^	0.013
	90 d	66.78 ± 3.26 ^D^	73.41 ± 1.03 ^C^	77.56 ± 0.76 ^B^	82.01 ± 2.11 ^A^	0.001
	120 d	68.44 ± 1.58 ^C^	77.43 ± 2.34 ^B^	82.37 ± 0.33 ^A^	84.65 ± 2.20 ^A^	0.001
Malondialdehyde MDA, nmol/mL	0 d	4.70 ± 1.22	4.09 ± 0.13	4.82 ± 0.78	4.61 ± 1.09	0.773
	30 d	4.44 ± 0.23	4.22± 0.08	4.06 ± 0.74	3.91 ± 0.45	0.537
	60 d	4.17 ± 0.29	3.97 ± 0.17	3.84 ± 0.13	3.69 ± 0.65	0.481
	90 d	3.98 ± 0.24 ^a^	3.69 ± 0.36 ^ab^	3.52 ± 0.32 ^ab^	3.31 ± 0.41 ^b^	0.047
	120 d	2.37 ± 0.75	2.85 ± 0.26	3.19 ± 0.63	2.85 ± 0.15	0.340
Glutathione peroxidase, U/mL	0 d	135.24 ± 5.61	134.69 ± 2.11	135.16 ± 1.34	140.39 ± 6.75	0.423
	30 d	137.12 ± 0.99 ^C^	155.21 ± 8.36 ^B^	163.07 ± 8.49 ^AB^	167.36 ± 3.09 ^A^	0.001
	60 d	146.01 ± 1.35 ^C^	166.83 ± 2.81 ^B^	170.11 ± 3.15 ^AB^	171.85 ± 1.29 ^A^	0.001
	90 d	151.28 ± 4.18 ^C^	171.69 ± 0.36 ^B^	177.47 ± 1.95 ^A^	182.31 ± 3.2 ^A^	0.001
	120 d	158.26 ± 5.91 ^C^	178.36 ± 2.49 ^B^	185.24 ± 1.67 ^B^	194.42 ± 3.33 ^A^	0.001
Total antioxidant capacity, U/mL	0 d	10.63 ± 0.12	10.63 ± 0.22	10.62 ± 0.05	10.67 ± 0.21	0.982
	30 d	10.59 ± 0.14	10.74 ± 0.03	10.71 ± 0.11	10.67 ± 0.14	0.491
	60 d	10.79 ± 0.04 ^A^	10.56 ± 0.21 ^B^	10.89 ± 0.03 ^A^	10.84 ± 0.06 ^A^	0.003
	90 d	10.85 ± 0.07	10.83 ± 0.08	10.89 ± 0.07	10.88 ± 0.05	0.063
	120 d	10.43 ± 0.18 ^B^	11.28 ± 0.09 ^A^	11.45 ± 0.21 ^A^	11.52 ± 0.21 ^A^	0.001

^A–D^ Different uppercase letters in the same row indicate highly significant differences (*p* < 0.01); ^a,b^ different lowercase letters indicate significant differences (*p* < 0.05). Identical letters indicate no significant difference. CK: control group; ML: 30% replacement; MT: 60% replacement; MC: 90% replacement. Values are presented as mean ± SEM of pen means (n = 3 pens per treatment, 7 lambs per pen).

**Table 7:** Effects of different replacement ratios of cotton stalks and sugar beet pulp silage on rumen fermentation parameters in Suffolk ram lambs.

Item	CK	MT	*p*-Value
pH	6.34 ± 0.19	6.18 ± 0.06	0.094
Acetic acid, mmol/L	50.76 ± 4.80	56.15 ± 0.72	0.118
Propionate, mmol/L	25.09 ± 0.76	26.67 ± 1.42	0.147
Butyric acid, mmol/L	16.12 ± 2.85	18.56 ± 1.14	0.226
Isobutyric acid, mmol/L	0.96 ± 0.09 ^b^	1.17 ± 0.10 ^a^	0.039
Isovaleric acid, mmol/L	1.15 ± 0.03	1.18 ± 0.06	0.452
Valeric acid, mmol/L	1.24 ± 0.03 ^b^	1.34 ± 0.03 ^a^	0.015
Caproic acid, mmol/L	0.46 ± 0.09	0.59 ± 0.08	0.105
Total volatile fatty acids, mmol/L	94.78 ± 6.57	102.48 ± 0.33	0.105

^a,b^ Different lowercase letters in the same row indicate significant differences (*p* < 0.05). Identical letters indicate no significant difference. CK: control group; MT: 60% replacement group. n = 6 lambs per treatment (2 lambs × 3 pens).

## Data Availability

The raw 16S rRNA gene sequencing data have been deposited in the NCBI Sequence Read Archive (SRA) under BioProject accession number PRJNA1507540. Other data supporting the findings of this study are available from the corresponding author upon reasonable request.

## Abbreviations

The following abbreviations are used in this manuscript:

ADG	Daily weight gain
ADFI	Average Daily dry matter intake
F:G	Feed-to-Gain Ratio
CSBPS	Cotton Stalk and Sugar Beet Pulp Silage
TMR	Total Mixed Ration
DM	Dry Matter
CP	Crude Protein
EE	Ether Extract
NDF	Neutral Detergent Fiber
ADF	Acid Detergent Fiber
ME	Metabolizable Energy
TP	Total Protein
ALB	Albumin
GLB	Globulin
TC	Total Cholesterol
TG	Triglycerides
HDL-C	High-Density Lipoprotein Cholesterol
LDL-C	Low-Density Lipoprotein Cholesterol
Cr	Creatinine
BUN	Blood Urea Nitrogen
GLU	Glucose
IgA	Immunoglobulin A
IgG	Immunoglobulin G
IgM	Immunoglobulin M
TNF-α	Tumor Necrosis Factor-alpha
IL-1β	Interleukin-1 beta
IL-4	Interleukin-4
SOD	Superoxide Dismutase
MDA	Malondialdehyde
GSH-Px	Glutathione Peroxidase
T-AOC	Total Antioxidant Capacity
VFA	Volatile Fatty Acids
TVFA	Total Volatile Fatty Acids
BCVFA	Branched-Chain Volatile Fatty Acids
SCFA	Short-Chain Fatty Acids
OTU	Operational Taxonomic Unit
PCoA	Principal Coordinate Analysis
LEfSe	Linear Discriminant Analysis Effect Size
LDA	Linear Discriminant Analysis
PERMANOVA	Permutational Multivariate Analysis of Variance
ELISA	Enzyme-Linked Immunosorbent Assay
GALT	Gut-Associated Lymphoid Tissue
GPR43	G Protein-Coupled Receptor 43
HDAC	Histone Deacetylase
Nrf2	Nuclear Factor Erythroid 2-Related Factor 2
NF-κB	Nuclear Factor Kappa B
ROS	Reactive Oxygen Species
